# Mitochondrial chromosome as a marker of animal migratory routes: DNA barcoding revealed Asian (non-African) origin of a tropical migrant butterfly *Junonia orithya* in south Israel

**DOI:** 10.3897/CompCytogen.v10i4.11085

**Published:** 2016-12-01

**Authors:** Vladimir A. Lukhtanov, Elena A. Pazhenkova, Asya V. Novikova

**Affiliations:** 1 Department of Karyosystematics, Zoological Institute of Russian Academy of Sciences, Universitetskaya nab. 1, 199034 St. Petersburg, Russia; 2 Department of Entomology, St. Petersburg State University, Universitetskaya nab. 7/9, 199034 St. Petersburg, Russia; 3 Department of Ecology, Evolution and Behavior, the Hebrew University of Jerusalem, Givat Ram, Berman bldg, 91904 Jerusalem, Israel

**Keywords:** barcode libraries, *COI*, Iran, Jordan, migration, Nymphalidae

## Abstract

The blue pansy *Junonia orithya* Linnaeus, 1758 (Lepidoptera, Nymphalidae) is widely distributed along the tropical areas of Africa, Asia and Australia. It is also known as a migrant species in the Levant. Here we record *Junonia orithya* in south Israel and provide a DNA-barcode-based evidence for its Asian (non-African) origin.

## Introduction

Despite its small size, the mitochondrial chromosome is a functionally important portion of the eukaryotic DNA (Taanman 1999). It is also an extremely useful genetic marker broadly used in genetic, phylogenetic, phylogeographic, biogeographic and taxonomic studies (Avise 2004, Talavera and Vila 2011, Lukhtanov et al. 2016). A relatively fast mutation rate and rapid sorting of mtDNA gene lineages, as well as absence (or at least rarity) of recombination usually result in high divergence of mitochondrial genomes among species and a comparatively small variance within species. For this reason, mtDNA-based species identification (so called DNA barcoding) has become a popular tool for identifying unknown specimens in terms of pre-existing classifications (Hebert et al. 2003, Chambers and Hebert 2016). This prompted DNA barcode databases (http://www.boldsystems.org/) and DNA reference barcode libraries (e.g. Dincă et al. 2011, 2015, Wilson et al. 2013).

Here we demonstrate how DNA barcode libraries result in an opportunity to study migration routes. Migration is a common phenomenon in animals (Dingle 2014, Chapman et al. 2015), but it is poorly studied in some groups, especially in insects. Within butterflies, with the exception of the relatively well studied monarch *Danaus plexippus* Linnaeus, 1758 (Brower 1995, Oberhauser et al. 2015) and the painted lady *Vanessa cardui* Linnaeus, 1758 (Talavera and Vila 2016), little is known about other species’ migratory routes.

The blue pansy *Junonia orithya* Linnaeus, 1758 belongs to a group of butterflies able to perform long-range migrations (Larsen 1984, Benyamini 2010). This species is widely distributed within the tropical areas of the Old World: in sub-Saharan Africa, Arabia, South and South-East Asia and Australia. It is known to be adapted to tropical environments and, according to Larsen (1984), could not normally survive winter even in the hottest spots of the Palaearctic region, such as the Jordan Valley in the Middle East. In south Iraq and south Iran, butterflies can be regularly observed in most months of the year, but in the central and northern parts of these countries they are less regular (Wiltshire 1957, Tshikolovets et al. 2014). Migrant individuals occur in Jordan (Larsen 1984, Katbeh-Bader et al. 2003, Benyamini 2010) and have been recently recorded in East Turkey (Biricik 2011). In Israel, records of *Junonia orithya* are known from the Jordan River valley in the north-east (Benyamini 2010). This distributional pattern fits well the hypothesis that specimens from Israel (as well as from the entire Middle East) might be connected to South Asia (and not to Africa, despite its geographical proximity), moving from south-east to north-west and finally reaching the most eastern parts of Israel.

On April 28, 2016 we collected a male of *Junonia orithya* in the southern tip of Israel in a small cultivated green patch near kibbutz Neot Smadar (30°02'40"N, 35°01'01"E, 409 m above sea level), enclosed by the Negev desert. Israel forms a biogeographic land bridge between Asia, Africa and Europe (Chipman et al. 2013); therefore the discovery of the blue pansy in the Negev, close to the African continent, raises a possibility of an African origin of *Junonia orithya* in Israel.

The “African” hypothesis seems to be plausible because animals and plants of African origin comprise a large group in the ecosystems of Israel, especially in the south where African elements are predominant (Goren and Ortal 1999). For example, butterfly fauna of south Israel includes such species of African origin as *Papilio saharae* Oberthür, 1879, *Euchloe aegyptiaca* Verity, 1911, *Euchloe falloui* Allard, 1867, *Epamera claucus* Butler, 1885, *Anthene amarah* (Guérin, 1849), *Azanus ubaldus* (Cramer, 1782), *Pseudophilotes abencerragus* (Pierret, 1837) and *Gomalia elma* (Trimen, 1862) (Benyamini 2010).

Here we tested the Asian versus African hypotheses by analyzing the barcode *COI* region of the mitochondrial genome. First we inspected all *COI* barcodes available from GenBank (https://www.ncbi.nlm.nih.gov/genbank/) and BOLD (http://www.boldsystems.org/index.php/Public_BarcodeIndexNumber_Home) and revealed that *Junonia orithya* comprised two genetically differentiated clusters of individuals. One cluster was represented by butterflies from Africa (Kenya, South Africa, Zimbabwe), and another cluster was represented by butterflies from Asia and Australia. Despite huge territory occupied by each of these clusters and despite the substantial divergence between the clusters, each group was found to be very homogenous with respect to the *COI* gene.

**Figure 1.**
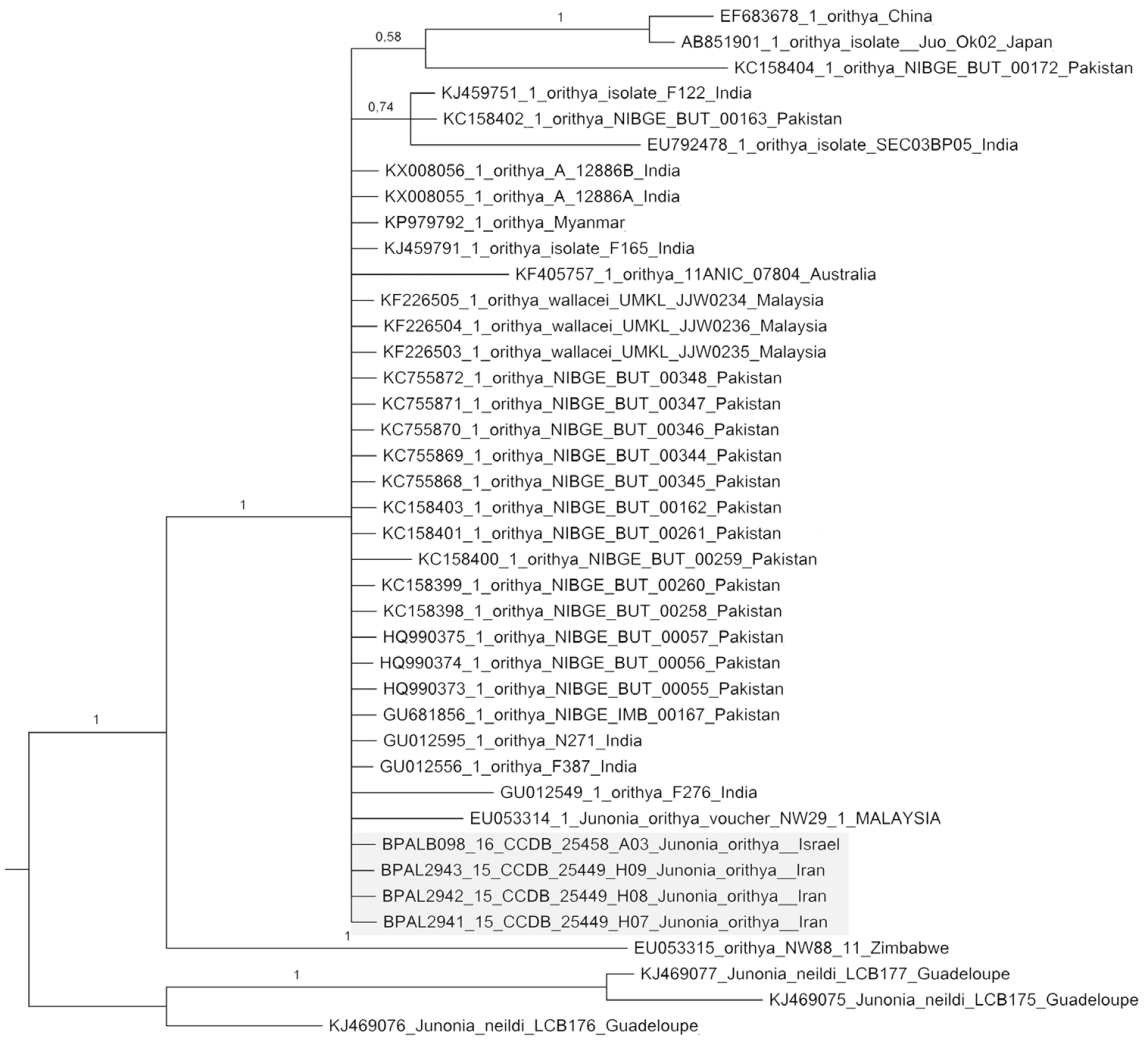
Bayesian tree of *Junonia orithya* samples based on analysis of the *cytochrome oxidase subunit I* (*COI*) gene (barcode region, 658 bp). Numbers at nodes indicate Bayesian posterior probability.

Then we obtained DNA barcodes for the sample from Neot Smadar, Israel (BPALB098-16, GenBank accession number: KY118822) and in three additional samples from Iran (from the collection of the Zoological Institute, St. Petersburg: BPAL2941-15 KY118824, BPAL2942-15 KY118825, BPAL2943-15 KY118823). These samples were processed as previously described (Lukhtanov et al. 2014, 2015). We used Bayesian Inference (MrBayes 3.1.2) as described previously (Vershinina and Lukhtanov 2010) to reconstruct a phylogenetic tree (Fig. 1). We used the published sequences of *Junonia neildi* Brévignon, 2004 (Gemmell et al. 2014) to root the tree (Fig. 1). Uncorrected *p*-distances were calculated manually based on direct comparison of sequences. In addition to our own sequences, only published data (one sample from Zimbabwe, Africa, one sample from Australia and 31 samples from Asia) (Kodandaramaiah and Wahlberg 2007, Zhang et al. 2008, Gaikwad et al. 2012, Ashfaq et al. 2013, Wilson et al. 2013, Hebert et al. 2013, Srirama et al. 2014, Yamada et al. 2014) were used for the phylogenetic inference (Fig. 1) and *p*-distance comparison.

The analysis revealed that the collected sample from south Israel was identical with eight samples from South Asia: with three samples from Malaysia (KF226505.1, KF226504.1, KF226503.1) and with five samples from Pakistan (GU681856.1, HQ990373.1, HQ990374.1, HQ990375.1, KC755868.1) (*p*-distance = 0%). It was also very close to other samples from Oriental and Australian regions as well as to the samples from Iran (Fig. 1) (*p*-distance from 0.2 to 1.3%). At the same time it differed by 19 nucleotide substitutions from the Zimbabwean sample (*p*-distance = 2.9%).

Thus, the genetic connectivity identified in our study supports the hypothesis that *Junonia orithya* may colonize Israel and Iran from tropical regions in Asia. Given that the species seem not to overwinter in the north of the Middle East, its sporadic presence suggests that these are immigrants, but precise origins of the specimens studied here could not be traced because of lack of phylogeographic structure within the analysed Asian populations. Since the species is common in certain areas in Arabia (Wiltshire 1957), that might be one of the most likely origin for these specimens. An African origin is not supported for these specimens and whether the Asian/Arabian origins are annually recurrent should be studied in detail with temporal series of immigrants. A wider African sampling, particularly from the northern hemisphere and especially from the nearest areas to Israel is also required for further testing the African hypothesis.
